# Identification of the Novel Interacting Partners of the Mammalian Target of Rapamycin Complex 1 in Human CCRF-CEM and HEK293 Cells

**DOI:** 10.3390/ijms15034823

**Published:** 2014-03-18

**Authors:** Hazir Rahman, Muhammad Qasim, Michael Oellerich, Abdul R. Asif

**Affiliations:** 1 Institute for Clinical Chemistry/UMG-Laboratories, University Medical Centre, Robert-Koch-Str. 40, Goettingen 37075, Germany; E-Mails: hazir.rahman@med.uni-goettingen.de (H.R.); muhammad.qasim@med.uni-goettingen.de (M.Q.); michael.oellerich@med.uni-goettingen.de (M.O.); 2 Department of Microbiology, Kohat University of Science and Technology, Kohat 26000, Pakistan

**Keywords:** mTORC1 purification, mTORC1 interacting proteins, hnRNP A2/B1 and dynamin 2

## Abstract

The present study was undertaken to identify proteins that interact with the mammalian target of rapamycin complex 1 (mTORC1) to enable it to carry out its crucial cell signaling functions. Endogenous and myc-tag mTORC1 was purified, in-gel tryptic digested and then identified by nano-LC ESI Q-TOF MS/MS analysis. A total of nine novel interacting proteins were identified in both endogenous and myc-tag mTORC1 purifications. These new mTORC1 interacting partners include heterogeneous nuclear ribonucleoproteins A2/B1, enhancer of mRNA decapping protein 4, 60S acidic ribosomal protein, P0, nucleolin, dynamin 2, glyceraldehyde 3 phosphate dehydrogenase, 2-oxoglutarate dehydrogenase, glycosyl transferase 25 family member 1 and prohibitin 2. Furthermore hnRNP A2/B1 and dynamin 2 interaction with mTORC1 was confirmed on immunoblotting. The present study has for the first time identified novel interacting partners of mTORC1 in human T lymphoblasts (CCRF-CEM) and human embryonic kidney (HEK293) cells. These new interacting proteins may offer new targets for therapeutic interventions in human diseases caused by perturbed mTORC1 signaling.

## 1. Introduction

The mammalian target of rapamycin (mTOR) is a serine threonine kinase that belongs to the phosphatidylinositol kinase-related protein kinase (PIKK) family, which regulates cell growth, cell proliferation and cell survival [1,2]. It was first coined as TOR in *Saccharomyces cerevisiae* and then found in higher eukaryotes as the specific target of rapamycin, a macrolide antibiotic produced by a soil bacterium, *Streptomyces hygroscopicus* [3]. Rapamycin, a potent immunosuppressive drug, inhibits mTOR by binding to its intracellular receptor, FK506 binding protein 12 (FKBP12), and interacts directly with the FKBP12-rapamycin binding (FRB) domain of mTOR [4–6]. mTOR kinase exists in two distinct multiprotein complexes, mTOR complex 1 (mTORC1) and mTOR complex 2 (mTORC2) [7,8]. Regulatory associated proteins of mTOR (raptor) and the rapamycin-insensitive companion of mTOR (rictor) are mutually exclusive in mTOR complexes [7,8]. mTORC1 has five components: mTOR, which is the catalytic subunit of the complex; regulatory-associated protein of mTOR (Raptor); G protein beta subunit-like (GβL), proline-rich AKT substrate 40 kDa (PRAS40) and DEP-domain-containing mTOR-interacting protein (Deptor) [7,9–11]. mTORC1 is a rapamycin-sensitive protein complex involved in energy and nutrient sensing, translation, transcription, autophagy, lipid biosynthesis and in the modulation of adaptive immunity [1,12–14]. mTOR kinase in mTORC1 executes a range of biological functions with the help of its interacting proteins [9]. It has been proposed that raptor might affect mTORC1 activity by regulating the assembly of the complex and by recruiting substrates for mTOR kinase [7,15]. The cellular localization of mTORC1 is reported to include neuronal membranes, mitochondria, endoplasmic reticulum, the Golgi apparatus, lysosomes and the nucleus [16], where mTORC1 might be associated with other interacting proteins. mTORC1 has a potential role in RNA synthesis and processing [17]. Heterogeneous nuclear ribonucleoproteins A2/B1 (hnRNP A2/B1) is the major protein present in the hnRNP RNA binding complex, involved in mRNA processing [18]. Moreover, the mTORC1 role in vesicular trafficking has been demonstrated earlier [19,20]. Dynamin 2 is a vesicular trafficking protein co-localized in the endomembrane compartments [21,22].

The present study was designed to identify the novel interacting partners of mTORC1 in human CCRF-CEM and human embryonic kidney (HEK293) cells. The immunoprecipitation/affinity purification followed by the mass spectrometric analysis strategy was used to identify the interacting proteins. Two interacting proteins (hnRNP A2/B1 and dynamin 2) were further verified by immunoblotting. These newly identified interacting proteins of mTORC1 may help broaden our understanding of mTORC1 signaling in human health and disease.

## 2. Results

The present study employed co-immunoprecipitation as an endogenous mTORC1 protein purification strategy to identify interacting proteins. In parallel, the myc-tag raptor component of mTORC1 pRK5 vector expression [7] in CCRF-CEM and HEK293 cells was used to purify the raptor binding complex of mTOR (mTORC1) and associated interacting partners using affinity column and monoclonal myc-tag antibody conjugated with agarose beads. In this method, the immunoprecipitation (IP) elute was relatively free from myc-tag antibody contamination, as the agarose beads were covalently linked with myc-tag antibody. The specificity of these interactions was insured by integrating appropriate purification controls (mock IP or antibody minus control).

### 2.1. Purification of Endogenous mTORC1

**Figure 1.**
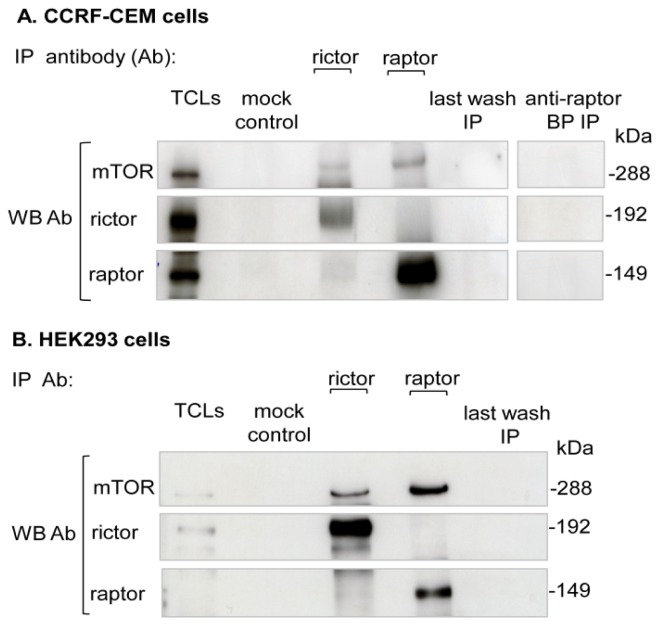
Endogenous mTORC1 purification: (**A**) CCRF-CEM or (**B**) HEK293 cells were grown for 48 h in complete medium and lysed as described in the Methods section. Endogenous mTOR complexes were immunopurified from total cell lysates (TCLs) using raptor or rictor antibodies. Mock (beads and whole cell lysates without antibody) or rictor immunoprecipitation (IP), last IP wash and anti-raptor blocking peptide (BP, which represents the epitope of the raptor antibody used for neutralization) were all included as purification controls. After immunopurification, the IP elutes were resolved on SDS-PAGE and then immunoblotted with corresponding antibodies. All experiments were independently repeated four times in each cell line (CCRF-CEM cells: *n* = 4; HEK293 cells: *n* = 4). WB in the figure represents Western blot.

In the present study, immunoprecipitated elutes of endogenous mTORC1specific purification were resolved on SDS-PAGE and immunoblotted individually with mTOR, raptor and rictor antibodies. In parallel, rictor IP elute was prepared and processed similarly to check for contamination of mTORC2 in raptor IP and *vice versa*. The mTOR signal was detected in both the raptor and rictor IP, which confirmed the successful co-immunoprecipitation of mTOR complexes. Immunoblotting with raptor antibody detected the raptor signal only in the raptor IP elute, whereas no rictor signal was detected in the raptor IP elute, indicating successful mTORC1-specific purification. Likewise, in the rictor IP elute, the rictor signal was detected, while no raptor signal was detected in the rictor IP elute, which confirmed specific mTORC2 purification. Mock IP or antibody minus control showed no cross-reactivity of raptor containing mTORC1 with the beads on immunoblot analysis. The raptor signal was not detected in either the last wash or in the anti-raptor blocking peptide (as a negative control to exclude any nonspecific lysate protein interaction with the raptor antibody) IP. This provided further evidence of the specific raptor containing mTORC1 purification (Figure 1A,B).

### 2.2. Purification of the Myc-Tag Raptor Component of mTORC1

**Figure 2.**
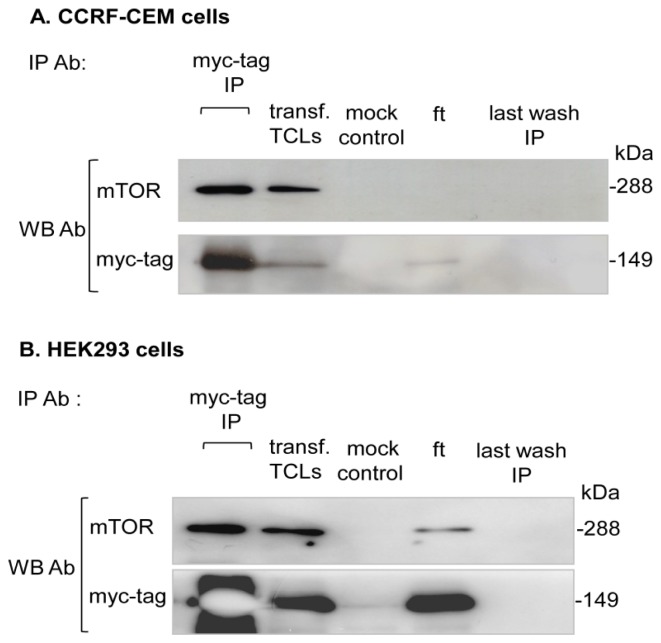
Purification of the myc-tag raptor component of mTORC1: (**A**) CCRF-CEM or (**B**) HEK293 cells were transiently transfected with myc-tag raptor pRK5 plasmid. After 48 h of transfection, cells were lysed, and the myc-tag raptor component of mTORC1 was immunoprecipitated with monoclonal myc-tag antibody conjugated beads. Transfected (transf.) HEK293 total cell lysates (TCLs), mock, flow through (fl) wash and last IP wash were incorporated as purification controls. IP elutes were resolved on the gel and immunoblotted with indicated antibodies. The myc-tag raptor component of the mTORC1 purification experiments was independently repeated twice, each in CCRF-CEM and HEK293 cells.

The total cell lysates (TCLs) of myc-tag raptor transfected cells were used to immunopurify the myc-tag raptor component of mTORC1. The immunoprecipitated elutes were resolved on SDS-PAGE and immunoblotted with indicated antibodies (Figure 2A,B).

### 2.3. Identification of mTORC1 Interacting Proteins Using Nano-LC ESI Q-TOF MS/MS Analysis

**Figure 3.**
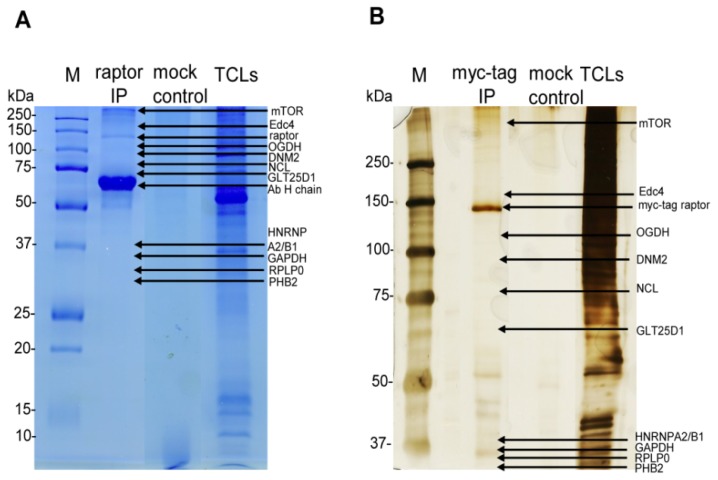
SDS-PAGE analysis of mTORC1 interacting proteins. (**A**) Endogenous mTORC1-specific purified elutes were prepared from CCRF-CEM cell lysates, resolved on SDS-PAGE and stained with Coomassie blue. The mock (negative) control sample was also run on the gel. After staining, the entire lane of protein bands was excised from the gel and tryptically digested for MS/MS analysis. Arrows on the gel indicate the approximate position of the identified proteins; (**B**) myc-tag mTORC1-specific purified elutes were prepared from HEK293 cells, separated on SDS-PAGE and stained with silver. Moreover, mock control samples were resolved on the gel. After staining, the entire lane of protein bands was excised from the gel and tryptically digested for MS/MS analysis. Arrows on the gel indicate the approximate position of the identified proteins.

After immunoblot confirmation of intact and purified mTORC1, the remaining IP elutes were run on the gel, stained with Coomassie blue (for endogenous mTORC1) and silver (for the myc-tag raptor component of mTORC1), as shown in Figure 3. After staining, the whole lane of raptor IP and mock control were excised from the gel for mass spectrometry analysis. All proteins identified from the mock controls were considered background contaminants and subtracted from the list of proteins identified from the raptor IP elution. A total of nine proteins common to both endogenous and myc-tag mTORC1 purifications were identified. The identified proteins and MS/MS spectral information are listed in Table 1 and in Supplementary Table S1 respectively.

**Figure 4.**
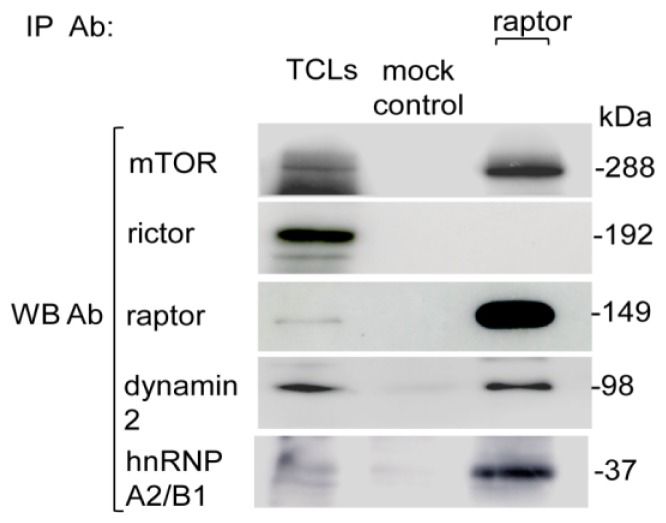
hnRNP A2/B1 and dynamin 2 interact with mTORC1. CCRF-CEM cells were lysed, and the raptor containing component of mTORC1 was immunoprecipitated using raptor antibody. The mTORC1-specific IP elute was resolved on SDS-PAGE and immunoblotted with corresponding antibodies, which showed substantial association of hnRNP A2/B1 and dynamin 2 with the raptor component of mTORC1.

Two novel interacting proteins’, hnRNP A2/B1 and dynamin 2, detection as mTORC1 interacting proteins were further verified by immunoblotting. The mTORC1-specific IP elute was immunoblotted with hnRNP A2/B1- and dynamin 2-specific antibodies (Figure 4). The hnRNP A2/B1 and dynamin 2 corresponding bands were detected in an mTORC1 immunoprecipitate, confirming the association of hnRNP A2/B1 and dynamin 2 with raptor containing mTORC1. This result is in close agreement with the mass spectrometric identifications (Table 1).

### 2.4. Functional Annotation and Protein-Protein Interaction Prediction

**Figure 5.**
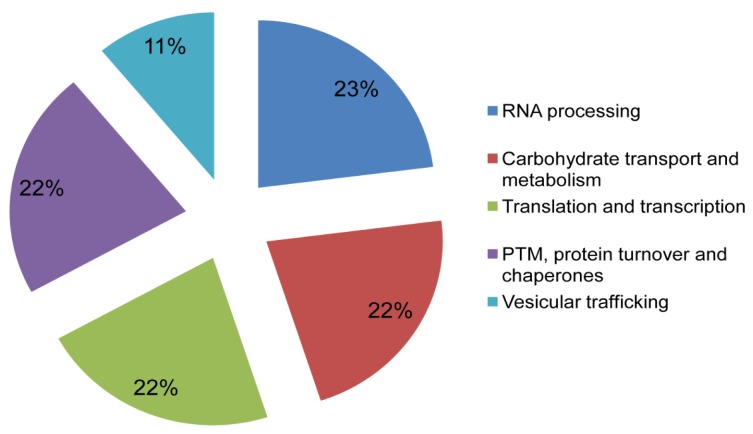
Functional annotation of newly identified mTORC1 interacting proteins. Putative biological functions of the identified proteins were assigned by using online functional annotation tools.

**Figure 6.**
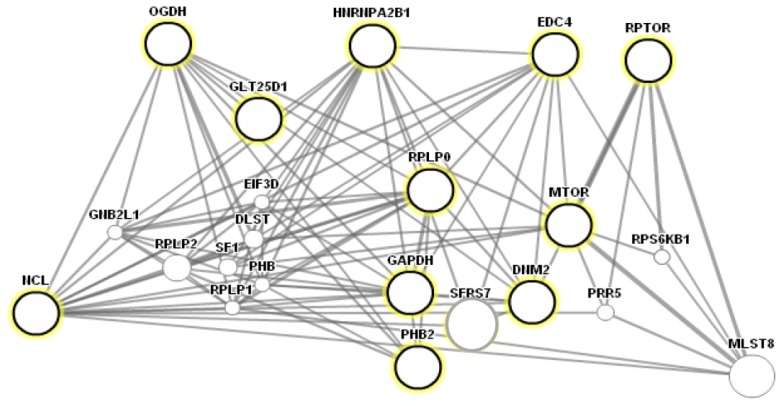
Biological interaction prediction of mTORC1 interacting proteins. An interaction map was predicted by using a web-based interface, GeneMANIA. The highlighted nodes represent mTOR or RPTOR (raptor) interacting proteins.

Putative cellular functions of the identified proteins were assigned as shown in Figure 5 by using Kognitor and universal protein (UniProt) [23,24] databases. Their functional annotations revealed that most proteins are involved in important cellular processes. Furthermore, GeneMANIA, a biological interaction prediction tool [25], was used to predict putative mTORC1 interaction with the newly identified proteins, as shown in Figure 6. Biological interaction prediction of the identified proteins revealed that these proteins mostly interacted with mTOR.

## 3. Discussion

The flow of cellular functions depends largely on signaling pathways that are regulated by specific protein-protein interactions [26]. These interactions often involve the assembly of large protein complexes containing many different protein kinases, their substrates and scaffold proteins [27]. mTOR kinase consists of two dynamic protein complexes, which differ in their composition, regulation and functions [7,8]. A growing body of literature involves new interacting protein partners of mTORC1 [7,9–11,28]. Although recent mass spectrometry-based methods are sufficient to identify the interacting partners of multiprotein complexes [29], a frequently faced problem is caused by the difficulty encountered in obtaining sufficient amounts of highly purified protein complexes. In this study, we report nine novel interacting partners of mTORC1, which are involved in important cellular functions.

Among the nine mTORC1 interacting proteins, two proteins, hnRNP A2/B1 and Edc4, were involved in mRNA processing. hnRNP A2/B1 is the major protein present in the hnRNP RNA binding complex [18]. The presence of hnRNP A2/B1 in mTORC1-specific purification upon immunoblotting further confirmed its interaction with mTORC1. Specific interactions of mTOR and S6K2 with hnRNPs are important in the regulation of cell proliferation [30]. Edc4 is an important protein involved in mRNA decapping, which is an essential step in the mRNA degradation [31].

Two interacting proteins identified in the category of translation and transcription were 60S acidic ribosomal protein P0 (RPLP0) and nucleolin (NCL). RPLP0 is a multifunctional protein required for efficient protein translation of the 60S ribosome [32]. mTORC1 has an important role in ribosome synthesis [33]. In this context, RPLP0 association with mTORC1 may be vital for ribosome biogenesis. NCL is involved in ribosome biogenesis, transport, cell proliferation and cell growth [34]. Insulin induces the NCL phosphorylation and increases ribosomal RNA transport [35]. mTORC1 is the crucial molecule in the regulation of ribosome biogenesis and is also stimulated by insulin and amino acids [36].

Dynamin 2 (DNM2), a large GTPase associated with vesicular trafficking [22], was identified as an interacting partner of mTORC1 in this study. DNM2 interacts and co-localizes with various proteins in the endomembrane compartments [21]. The localization of mTORC1 in the endoplasmic reticulum and the Golgi apparatus and its role in the membrane trafficking were demonstrated earlier [19].

In the category of carbohydrate transport and metabolism, both glyceraldehyde 3 phosphate dehydrogenase (GAPDH) and 2-oxoglutarate dehydrogenase (2-OADH) were identified as interacting partners. The role of mTORC1 is already implicated in glycolytic flux and energy sensing [33].

Furthermore, we identified glycosyltransferase 25 family member 1 (GLT25D1) and prohibitin 2 (PHB2) as mTOR interacting proteins, involved in post-translational modification, protein turnover and chaperone functions. mTORC1 regulates these biological processes by post-translational modification, especially via phosphorylation [1].

In general, there are three possibilities for mTORC1 interactions with the newly identified interacting proteins: (a) these interacting proteins may act as a direct substrate of mTORC1, and phosphorylation of such proteins via mTORC1 could be part of their regulatory mechanism [36]; (b) the interacting proteins may bind to mTORC1 and enhance or inhibit the mTORC1 kinase activity [9,10]; or (c) mTORC1 may compete with the inhibitor or activator of these proteins.

## 4. Experimental Section

### 4.1. Antibodies and Reagents

Antibodies and reagents were obtained from the following sources: the raptor antibody (catalogue No. 09-217) was from Millipore, Schwalbach Germany. mTOR (catalogue No. 2972) and myc-tag (catalogue No. 2272) antibodies were from Cell Signaling Technology, Danvers, MA, USA. The rictor antibody (catalogue No. A300-459A) was from Bethyl Laboratories, Montgomery, TX, USA. hnRNP A2/B1 (catalogue No. sc-32316) and dynamin 2 (catalogue No. sc-6400) was from Santa Cruz Biotechnology, Dallas, TX, USA. Horseradish peroxidase (HRP) labelled secondary antibodies were from Bio-Rad, Munich, Germany. CHAPS buffer was from Applichem, Darmstadt, Germany. The complete protease and phosphatase inhibitor cocktail were from Roche, Mannheim, Germany. Dithiothreitol (DTT), trypsin, trifluoroacetic acid (TFA), formic acid (FA), acetonitrile (ACN) and ammonium bicarbonate (AMBIC) were from Sigma-Aldrich, Steinheim, Germany. RPMI-1640, DMEM, phosphate buffer saline (PBS), penicillin and streptomycin were from PAA Laboratories, Colbe, Germany. The myc-tag Co-IP kit was from Thermo Scientific Pierce, Rockford, IL, USA. The myc-tag raptor pRK5 plasmid was a kind gift of Dr. Doss Sarbassove (The University of Texas, Austin, TX, USA).

### 4.2. Cell Culture

Human T lymphoblasts (CCRF-CEM) and human embryonic kidney (HEK293) cells were purchased from DSMZ (German collection of microorganisms and cell cultures, Braunschweig, Germany). CCRF-CEM and HEK293 cells were grown in 75-cm^2^ culture flasks (Sarstedt, Nuemberecht, Germany) and maintained in culture at 37 °C in 95% humidity, 20% O_2_ and 5% CO_2_. RPMI-1640 and DMEM culture medium supplemented with 10% fetal calf serum, 100,000 U/L penicillin and 100 μg/L streptomycin (Biochrome, Berlin, Germany) were used to grow CCRF-CEM and HEK293 cells, respectively. Cell confluency was regularly checked, and the medium was changed accordingly.

### 4.3. Cell Lysis and Endogenous mTORC1 Purification

Cells (60 million) were rinsed with cold PBS and lysed on ice-cold CHAPS buffer lacking NaCl (40 mM HEPES [pH 7.4], 0.3% CHAPS, protease and phosphatase inhibitors) to isolate mTOR complexes [9]. Cell debris was removed from the lysates by centrifugation at 13,000 rpm for 15 min at 4 °C, followed by pre-clearing with dynabeads G. Antibodies for immunoprecipitation (IP) and co-immunoprecipitation (Co-IP) were added to the lysate (1 mg/mL) and incubated for 30 min at 4 °C. Dynabeads G (40 μL) were added to the antibody and lysate mixture and incubated overnight at 4 °C. Immunoprecipitation of specific rictor containing mTORC2 using rictor antibody was incorporated as a negative control to validate the purity of specific raptor-containing mTORC1. The mock control (beads and whole cell lysates without adding antibody) was used to exclude the false interaction of lysate proteins with the dynabeads. In addition, blocking peptide (BP) was synthesized (Seq Laboratories, Goettingen, Germany) and represents the epitope of a raptor antibody. BP was incorporated as a negative IP control (only for CCRF-CEM cells) to exclude any nonspecific lysate protein interaction with the antibody. For the blocking peptide IP control, 30 μg BP was added to 3 μg raptor antibody and incubated overnight at 4 °C. After incubation, the antibody and blocking peptide mixture was added to the cell lysate containing the dynabeads and then incubated overnight at 4 °C. The mock control was also incubated overnight at 4 °C. Immunoprecipitates and mock controls were washed once with CHAPS buffer lacking NaCl and three times with CHAPS buffer containing 150 mM NaCl. Washes were saved for parallel runs with IP elute on immunoblotting. Samples were eluted in 2× Laemmli buffer at 95 °C for 10 min and resolved on 6% SDS-PAGE. For experiments with cell lysates, Triton X-100 containing lysis buffer (20 M Tris-HCl [pH 7.5], 150 mM NaCl, 1 mM Na_2_EDTA, 1 mM EGTA, 1% Triton X-100, 2.5 mM sodium pyrophosphate, 1 mM beta-glycerophosphate, 1 mM Na_3_VO_4_, 1 μg/mL leupeptin, protease and phosphatase inhibitors) was used.

### 4.4. Mammalian Cells Transfection and Myc-Tag mTORC1 Purification

CCRF-CEM and HEK293 (8 million each) cells were seeded in 6-well plates for myc-tag raptor pRK5 transfection. Lipofectamine LTX and Plus reagent were used according to the vendor’s recommendations (Invitrogen, Darmstadt, Germany). Briefly, 3 μg of myc-tag raptor pRK5 plasmid and 3 μL of Plus reagent were added to Opti MEM and incubated for five minutes. Then, 4 μL of Lipofectamine LTX were added to the mixture that was then incubated for 30 min at room temperature. The mixture was added to the cells and incubated at 37 °C in a CO_2_ incubator for 48 h. Cells were rinsed with cold PBS and lysed on ice-cold CHAPS buffer lacking NaCl to isolate mTOR-containing complexes. Cell lysates were separated from insoluble cell debris by centrifugation at 13,000 rpm for 15 min at 4 °C. A myc-tag Co-IP kit was used according to the manufacturer’s instructions (Thermo Scientific Pierce, Rockford, IL, USA). Briefly, lysates (1 mg/mL) were added to the spin column followed by the addition of myc-tag monoclonal antibody conjugated beads and incubated overnight at 4 °C. Mock controls were run as a negative control. Immunoprecipitates were washed once with CHAPS buffer lacking NaCl and three times with CHAPS buffer containing NaCl and the washes saved. The samples were eluted with glycine buffer (pH 2.8), neutralized by the addition of 1 Mol Tris (pH 9.5) and processed for SDS-PAGE.

### 4.5. SDS-PAGE and Immunoblot Analysis

Proteins elutes were resolved on 6% SDS-PAGE and blotted onto polyvinylidene difluoride (PVDF) membrane (Millipore, Schwalbach, Germany) using the semidry Trans-Blot Semi Dry cell system (Bio-Rad, Munich, Germany) for 30 min at 17 V in a transfer buffer (192 mM glycine, 10% methanol, 25 mM Tris-HCl (pH 8.3). The membrane was blocked with 5% skimmed milk powder prepared in TBS-T buffer (50 mmol/L Tris-HCl (pH 7.5), 200 mmol/L NaCl, 0.05% Tween 20) for one hour at room temperature and washed three times with TBS-T buffer. Primary antibody was added for overnight incubation at 4 °C. After three washes with TBS-T, the membrane was incubated in HRP-conjugated secondary antibodies for one hour at room temperature and then washed three times in TBS-T for 10 min each. The signals on the blot were detected using enhanced chemiluminescent (ECL) reagent (GE Healthcare, Buckinghamshire, UK) and then developed on Amersham Hyperfilm (GE Healthcare, Buckinghamshire, UK). Signal intensities for each immunoblot were quantified using the Lab Image software version 2.71 (Kapelan, Leipzig, Germany).

### 4.6. Protein Visualization and In-Gel Digestion of Proteins

Following confirmation of mTORC1-specific purification on immunoblotting, the remaining IP elutes were run on the 12.5% SDS-PAGE and stained with colloidal Coomassie blue (Carl Roth, Karlsruhe, Germany) or silver nitrate, as previously described [37]. After staining, the entire lane of raptor IP and the mock control were excised from the gel (30 to 40 slices) and tryptically digested for MS/MS analysis, as described in [38], with some modifications. Briefly, excised gel spots were destained and washed with ACN (50%) and AMBIC (100 mM), followed by drying in a vacuum centrifuge (UNIVAPO, uniEquip, Matinsried, Germany). The dried gel pieces were digested with trypsin containing digestion buffer (0.1 μg/μL trypsin, 1 M CaCl_2_, 1 M AMBIC (pH 7.4)) for 45 min on ice. The excess amount of trypsin solution was replaced by the same volume of 100 mM of AMBIC without trypsin and incubated overnight at 37 °C. The peptides were extracted with increasing concentrations of ACN and TFA and dried by vacuum centrifugation.

### 4.7. Peptide Sequence Analysis by Nano-LC ESI Q-TOF MS/MS and Database Search

The peptides were reconstituted in an aqueous solution of 0.1% formic acid. For LC-MS/MS analysis, 1 μL of the reconstituted peptide sample was introduced on to two consecutive C18-reversed phase chromatography columns (C18 pepMap100 nano-analytical column: 75 μm × 15 cm; 3 μm particle size; and C18 pepMap: 300 μm × 5 mm; 5 μm particle size; LC Packings, Emsdetten, Germany) using a CapLC nano-flow auto sampler (Waters, Eschborn, Germany). The single sample run time was set for 60 min. Protein peptides were chromatographically resolved and analyzed on a Q-TOF Ultima Global mass spectrometer (Micromass, Manchester, UK) equipped with a positive ion mode ESI Z-spray source, as described by [39]. The multiple charged peptide ions were automatically marked and selected in the quadrupole, fragmented in the hexapole collision cell, and their fragment patterns were analyzed by TOF. The data were acquired with the MassLynx (v 4.0) software (Waters Corporation Milford, MA, USA) on a Windows NT PC and processed using ProteinLynx Global Server (PLGS, v 2.2, Micromass, Manchester, UK) as PKL (peak list) under the following settings: electrospray, centroid, 80% with minimum peak width, 4 channel, medium deisotoping with a 3% threshold, no noise reduction and no smoothing. The peak lists (PKLs) were examined using the MASCOT (http://www.matrixscience.com, Matrix Science, Boston, MA, USA) algorithm against the Swiss-Prot version 57.0 (525,997 sequences; 185,874,894 residues) and NCBInr (14,269,787 sequences; 4,888,943,253 residues) protein databases. The acquired data were compared to the whole database with search parameters set as follows: enzyme, trypsin; allowance of up to one missed cleavage peptide; mass tolerance ±0.5 Da and MS/MS tolerance ±0.5 Da; modifications of methionine oxidation and cysteine carbamidomethylation when appropriate, with auto hits allowed and only significant hits to be reported. The proteins were identified on the basis of two or more peptides whose ion scores exceeded the threshold, *p* < 0.05, which indicated the 95% confidence level for these matched peptides. Proteins were identified from the database on the basis of at least two or more peptides whose ion scores exceeded the threshold, *p* < 0.05, which indicated the 95% confidence level for the matched peptides. The nano-ESI LC Q-TOF MS/MS analysis was repeated independently from eight independent preparations for endogenous mTORC1 purification and four independent preparations for myc-tag mTORC1 purifications to ensure the reproducibility of the purification and the accuracy of the protein identification.

### 4.8. Functional Annotation and Protein-Protein Interaction Prediction

Functional annotation of all newly identified proteins was provided by matching their accession number and obtained amino acid sequences using the universal protein (UniProt) [24] and NCBI Kognitor [23] databases. Moreover, *in silico* protein-protein interaction prediction was obtained from a web-based interface, GeneMANIA (http://www.genemania.org), which is a biological interaction prediction tool [25] that can be used to predict mTORC1 interaction with the newly identified proteins.

## 5. Conclusions

In the present study, we identified nine new interacting proteins of mTORC1 using both endogenous purification and exogenous myc-tag purification strategies. Functional understanding of these new interacting proteins may be helpful in providing targets for new therapeutic interventions for human diseases in which mTORC1 signaling may be perturbed.

## Supplementary Information



## Figures and Tables

**Table 1.** Novel interacting partners of the raptor component of mTORC1 identified using nano-LC ESI Q-TOF MS/MS analysis.

Accession No.	Protein Name	Mass (kDa)	Protein Function	c Mascot Score	d pI	ePeptide Matches (sequences)
Q8N122	a Raptor	149	Translation and transcription	450	6.43	14 (11)
P42345	b mTOR	288	Translation and transcription	254	6.73	18 (11)
P22626	hnRNPA2/B1	37	mRNA processing	106	8.97	3 (3)
Q6P2E9	Edc4	151.6	mRNA processing	92	5.55	3 (3)
P05388	RPLP0	34.3	Translation and transcription	105	5.41	3 (3)
P19338	Nucleolin	76.5	Translation and transcription	133	4.6	8 (5)
P50570	DNM2	98	Vesicular trafficking	63	7.04	5 (3)
P04406	GAPDH	36	Carbohydrate transport and metabolism	114	8.57	7 (4)
Q8NBJ5	GLT25D1	71.5	Post-translational modification, protein turnover and chaperone functions	148	6.85	6 (6)
Q99623	PHB2	33.2	Post-translational modification, protein turnover and chaperone functions	147	9.83	3 (3)

a and b are the known interacting partners of mTORC1;

c Mascot score: >42 indicates identity or extensive homology (*p* < 0.05);

d pI: isoelectric pH;

e proteins’ peptides were identified by ESI Q-TOF MS/MS analysis from the Coomassie blue or silver stained gel of specific mTORC1 purification material prepared from CCRF-CEM and HEK293 cells. All endogenous mTORC1 purification experiments were repeated four times in each cell line (CCRF-CEM cells *n* = 4; HEK293 cells, *n* = 4). The myc-tag purification of mTORC1 was repeated twice (CCRF-CEM cells, *n* = 2; HEK293 cells, *n* = 2) to ensure accurate protein identification after the endogenous mTORC1 purification.
