# Supplementary material for: Identification of the Novel Interacting Partners of the Mammalian Target of Rapamycin Complex 1 in Human CCRF-CEM and HEK293 Cells
Source: Int J Mol Sci. 2014 Mar 18;15(3):4823–36. doi: 10.3390/ijms15034823 (PMC3975426; doi:10.3390/ijms15034823)
Supplement: Supplementary file 1 [file ijms-15-04823-s001.pdf]

Supplementary Information

Table S1. MS/MS data for mTORC1 interacting proteins identified by ESI Q-TOF MS/MS analysis.

**Protein Name**	[a] **Peptides Matches (sequences)**	[b] **MS/MS Analysis**
Raptor	14 (11)	1 MESEMLQSPL LGLGEEDEAD LTDWNLPLAF MKKRHCEKIE GSKSLAQSWR<br>51 MKDRMKTVSV ALVLCLNVGV DPPDVVKTTP CARLECWIDP LSMGPQK**ALE**<br>101 **TIGANLQK**QY ENWQPRARYK QSLDPTVDEV KKLCTSLRRN AKEERVLFHY<br>151 NGHGVPRPTV NGEVWVFNKN YTQYIPLSIY DLQTWMGSPS IFVYDCSNAG<br>201 LIVKSFKQFA LQREQELEVA AINPNHPLAQ MPLPPSMKNC IQLAACEATE<br>251 LLPMIPDLPA DLFTSCLTTP IKIALRWFCM QKCVSLVPGV TLDLIEKIPG<br>301 RLNDRRTPLG ELNWIFTAIT DTIAWNVLPR DLFQKLFR**QD LLVASLFRNF**<br>351 **LLAER**IMRSY NCTPVSSPRL PPTYMHAMWQ AWDLAVDICL SQLPTIIEEG<br>401 TAFRHSPFFA EQLTAFQVWL TMGVENRNPP EQLPIVLQVL LSQVHRLRAL<br>451 DLLGRFLDLG PWAVSLALSV GIFPYVLKLL QSSARELRPL LVFIWAKILA<br>501 VDSSCQADLV KDNGHKYFLS VLADPYMPAE HRTMTAFILA VIVNSYHTGQ<br>551 EACLQGNLIA ICLEQLNDPH PLLRQWVAIC LGR**IWQNFDS AR**WCGVRDSA<br>601 HEK**LYSLLSD PIPEVR**CAAV FALGTFVGNS AERTDHSTTI DHNVAMMLAQ<br>651 LVSDGSPMVR KELVVALSHL VVQYESNFCT VALQFIEEEK NYALPSPATT<br>701 EGGSLTPVRD SPCTPRLR**SV SSYGNIR**AVA TARSLNKSLQ NLSLTEESGG<br>751 AVAFSPGNLS TSSSASSTLG SPENEEHILS FETIDKMRRA SSYSSLNSLI<br>801 GVSFNSVYTQ IWRVLLHLAA DPYPEVSDVA MK**VLNSIAYK** ATVNARPQRV<br>851 LDTSSLTQSA PASPTNKGVH IHQAGGSPPA SSTSSSSLTN DVAKQPVSRD<br>901 LPSGRPGTTG PAGAQYTPHS HQFPRTRKMF DK**GPEQTADD ADDAAGHK**SF<br>951 ISATVQTGFC DWSARYFAQP VMKIPEEHDL ESQIRKEREW RFLRNSRVRR<br>1001 QAQQVIQKGI TR**LDDQIFLN R**NPGVPSVVK FHPFTPCIAV ADKDSICFWD<br>1051 WEKGEKLDYF HNGNPRYTRV TAMEYLNGQD CSLLLTATDD GAIRVWKNFA<br>1101 DLEKNPEMVT AWQGLSDMLP TTRGAGMVVD WEQETGLLMS SGDVRIVR**IW**<br>1151 **DTDR**EMKVQD IPTGADSCVT SLSCDSHR**SL IVAGLGDGSI R**VYDRRMALS<br>1201 ECRVMTYREH TAWVVKASLQ KRPDGHIVSV SVNGDVRIFD PRMPESVNVL<br>1251 QIVKGLTALD IHPQADLIAC GSVNQFTAIY NSSGELINNI KYYDGFMGQR<br>1301 VGAISCLAFH PHWPHLAVGS NDYYISVYSV EKRVR<br>**Start-End Observed Mr(expt) Mr(calc) Delta Miss Sequence**<br>**98 -108 579.3115 1156.60841156.6452 -0.0367 0 U K.ALETIGANLQK.Q**<br>**98 -108 579.3149 1156.6152 1156.6452 -0.0299 0 U K.ALETIGANLQK.Q**<br>**339 -348 581.3126 1160.6106 1160.6553 -0.0447 0 R.QDLLVASLFR.N**<br>**349 -355 431.7291 861.4436 86 1.4708 -0.0272 0 R.NFLLAER.I**<br>**584 -592 568.7628 1135.5110 1135.5410 -0.0300 0 U R.IWQNFDSAR.W**<br>**604 -616 751.3935 1500.7724 1500.8188 -0.0463 0 U K.LYSLLSDPIPEVR.C**<br>**604 -616 751.3962 1500.7778 1500.8188 -0.0409 0 U K.LYSLLSDPIPEVR.C**<br>**719 -727 491.7391 981.4636 981.4879 -0.0243 0 U R.SVSSYGNIR.A**<br>**833 -840 454.2522 906.4898 906.5174 -0.0276 0 U K.VLNSIAYK.A**<br>**933 -948 533.2261 1596.6565 1596.6652 -0.0087 0 U K.GPEQTADDADDAAGHK.S**<br>**1013 -1021 567.2834 1132.5522 1132.5877 -0.0354 0 U R.LDDQIFLNR.N**<br>**1013 -1021 567.2839 1132.5532 1132.5877 -0.0344 0 U R.LDDQIFLNR.N**<br>**1149 -1154 403.1828 804.3510 804.3766 -0.0256 0 U R.IWDTDR.E**<br>**1179 -1191 629.3464 1256.6782 1256.7088 -0.0306 0 UR.SLIVAGLGDGSIR.V**<br>**MS/MS Fragmentation of SLIVAGLGDGSIR**

Table S1. *Cont.*

Protein Name	[a] Peptides Matches (sequences)	[b] MS/MS Analysis
Raptor	14 (11)	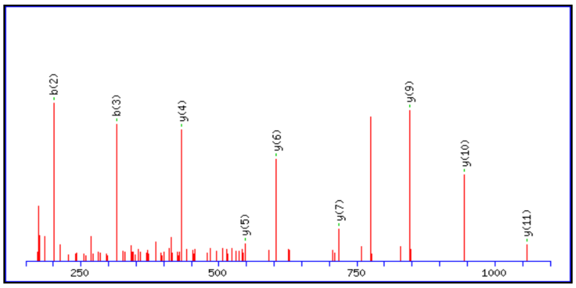
mTOR	18 (11)	1 MLGTGPAAAT TAATTSSNVS VLQQFASGLK SRNEETRAKA AKELQHYVTM<br>51 ELREMSQEES TRFYDQLNHH IFELVSSSDA NERKGGILAI ASLIGVEGGN<br>101 ATRIGRFANY LRNLLPSNDP VVMEMASKAI GRLAMAGDTF TAEYVEFEVK<br>151 RALEWLGADR NEGRRHAAVL VLRELAISVP TFFFQQVQPF FDNIFVAVWD<br>201 PKQAIREGAV AALRACLILT TQREPKEMQK PQWYRHTFEE AEKGFDETLA<br>251 KEKGMNRDDR IHGALLILNE LVRISSMEGE RLREEMEEIT QQQLVHDKYC<br>301 KDLMGFGTKP RHITPFTSFQ AVQPQQSNAL VGLLGYSSHQ GLMGFGTSPS<br>351 PAKSTLVESR CCRDLMEEKF DQVCQWVLKC RNSKNSLIQM TILNLLPRLA<br>401 AFRPSAFTDT QYLQDTMNHV LSCVKKEKER TAAFQALGLL SVAVRSEFKV<br>451 YLPRVLDIIR AALPPKDFAH KRQKAMQVDA TVFTCISMLA RAMGPGIQQD<br>501 IKELLEPMLA VGLSPALTAV LYDLSRQIPQ LKKDIQDGLL KMLSLVLMHK<br>551 PLRHPGMPKG LAHQLASPGL TTLPEASDVG SITLALR**TLG SFEFEGHSLT**<br>601 **QFVR**HCADHF LNSEHKEIRM EAARTCSRLL TPSIHLISGH AHVVSQTAVQ<br>651 VVADVLSKLL VVGITDPDPD IRYCVLASLD ERFDAHLAQA ENLQALFVAL<br>701 NDQVFEIREL AICTVGRLSS MNPAFVMPFL RKMLIQILTE LEHSGIGRIK<br>751 EQSARMLGHL VSNAPRLIRP YMEPILKALI LKLKDPDPDP NPGVINNVLA<br>801 TIGELAQVSG LEMRKWVDEL FIIIMDMLQD SSLLAKRQVA LWTLGQLVAS<br>851 TGYVVEPYRK YPTLLEVLLN FLKTEQNQGT RREAIRVLGL LGALDPYKHK<br>901 VNIGMIDQSR **DASAVSLSES K**SSQDSSDYS TSEMLVNMGN LPLDEFYPAV<br>951 SMVALMRIFR DQSLSHHHTM VVQAITFIFK SLGLKCVQFL PQVMPTFLNV<br>1001 IRVCDGAIRE FLFQQLGMLV SFVKSHIRPY MDEIVTLMRE FWVMNTSIQS<br>1051 TIILLIEQIV VALGGEFKLY LPQLIPHMLR VFMHDNSPGR IVSIKLLAAI<br>1101 QLFGANLDDY LHLLLPPIVK **LFDAPEAPLP SR**KAALETVD RLTESLDFTD<br>1151 YASRIIHPIV R**TLDQSPELR** STAMDTLSSL VFQLGKKYQI FIPMVNKVLV<br>1201 RHRINHQRYD VLICRIVK**GY TLADEEEDPL IYQHR**MLRSG QGDALASGPV<br>1251 ETGPMKKLHV STINLQKAWG AARRVSKDDW LEWLRRLSLE LLKDSSSPSL<br>1301 RSCWALAQAY NPMARDLFNA AFVSCWSELN EDQQDELIRS IELALTSQDI<br>1351 AEVTQTLLNL AEFMEHSDKG PLPLRDDNGI VLLGERAAKC RAYAKALHYK<br>1401 ELEFQK**GPTP AILESLISIN NK**LQQPEAAA GVLEYAMKHF GELEIQATWY<br>1451 EKLHEWEDAL VAYDKKMDTN KDDPELMLGR MRCLEALGEW GQLHQQCCEK<br>1501 WTLVNDETQA KMARMAAAAA WGLGQWDSME EYTCMIPRDT HDGAFYRAVL<br>1551 ALHQDLFSLA QQCIDKARDL LDAELTAMAG ESYSRAYGAM VSCHMLSELE<br>1601 EVIQYKLVPE RREIIRQIWW ERLQGCQRIV EDWQKILMVR SLVVSPHEDM<br>1651 RTWLKYASLC GKSGRLALAH K**TLVLLLGVD PSR**QLDHPLP TVHPQVTYAY<br>1701 MKNMWKSARK IDAFQHMQHF VQTMQQQAQH AIATEDQQHK QELHKLMARC<br>1751 FLKLGEWQLN LQGINESTIP KVLQYYSAAT EHDRSWYKAW HAWAVMNFEA<br>1801 VLHYKHQNQA RDEKKKLRHA SGANITNATT AATTAATATT TASTEGSNSE<br>1851 SEAESTENSP TPSPLQKKVT EDLSKTLLMY TVPAVQGFFR SISLSR**GNNL**<br>1901 **QDTLR**VLTLW FDYGHWPDVN EALVEGVKAI QIDTWLQVIP QLIARIDTPR

Table S1. *Cont.*

Protein Name	[a] Peptides Matches (sequences)	[b] MS/MS Analysis
mTOR	18 (11)	1951 PLVGRLIHQL LTDIGRYHPQ ALIYPLTVAS KSTTTARHNA ANKILKNMCE<br>2001 HSNTLVQQAM MVSEELIRVA ILWHEMWHEG LEEASRLYFG ERNVKGMFEV<br>2051 LEPLHAMMER GPQTLK**ETSF NQAYGR**DLME AQEWCRKYMK SGNVKDLTQA<br>2101 WDLYYHVFRR ISKQLPQLTS LELQYVSPKL LMCRDLELAV PGTYDPNQPI<br>2151 IR**IQSIAPSL QVITSK**QRPR KLTLMGSNGH EFVFLLKGHE DLRQDERVMQ<br>2201 LFGLVNTLLA NDPTSLRKNL SIQRYAVIPL STNSGLIGWV PHCDTLHALI<br>2251 RDYREKKKIL LNIEHRIMLR MAPDYDHLTL MQK**VEVFEHA VNNTAGDDLA**<br>2301 **K**LLWLKSPSS EVWFDRRTNY TRSLAVMSMV GYILGLGDRH PSNLMLDRLS<br>2351 GKILHIDFGD CFEVAMTREK FPEKIPFRLT RMLTNAMEVT GLDGNYRITC<br>2401 HTVMEVLREH KDSVMAVLEA FVYDPLLNWR LMDTNTKGNK RSRTRTDSYS<br>2451 AGQSVEILDG VELGEPAHKK TGTTVPESIH SFIGDGLVKP EALNKKAIQI<br>2501 INRVRDKLTG RDFSHDDTLD VPTQVELLIK QATSHENLCQ CYIGWCPFW<br>**Start-End Observed Mr(expt) Mr(calc) Delta Miss Sequence**<br>**588 – 604 652.3008 1953.8806 1953.9585 -0.0779 0 U R.TLGSFEFEGHSLTQFVR.H**<br>**911 – 921 547.2456 1092.4766 1092.5299 -0.0532 0 U R.DASAVSLSESK.S**<br>**911 – 921 547.2457 1092.4768 1092.5299 -0.0530 0 U R.DASAVSLSESK.S**<br>**1121 – 1132 656.8223 1311.6300 1311.6823 -0.05220 U K.LFDAPEAPLPSR.K**<br>**1121 – 1132 656.8223 1311.6300 1311.6823 -0.05220 U K.LFDAPEAPLPSR.K**<br>**1162 – 1170 529.7536 1057.4926 1057.5404 -0.04770 U R.TLDQSPELR.S**<br>**1162 – 1170 529.7536 1057.4926 1057.5404 -0.0477 0 U R.TLDQSPELR.S**<br>**1219 – 1235 683.6269 2047.8589 2047.9487 -0.0898 0 U K.GYTLADEEEDPLIYQHR.M**<br>**1219 – 1235 683.6269 2047.8589 2047.9487 -0.0898 0 U K.GYTLADEEEDPLIYQHR.M**<br>**1407 – 1422 833.9456 1665.8766 1665.9301 -0.0535 0 U K.GPTPAILESLISINNK.L**<br>**1672 – 1683 641.8618 1281.7090 1281.7656 -0.0566 0 U K.TLVLLLGVDPSR.Q**<br>**1897 – 1905 515.7442 1029.4738 1029.5203 -0.04650 U R.GNNLQDTLR.V**<br>**1897 – 1905 515.7442 1029.4738 1029.5203 -0.0465 0 U R.GNNLQDTLR.V**<br>**2067 – 2076 586.7448 1171.4750 1171.5258 -0.0507 0 U K.ETSFNQAYGR.D**<br>**2153 – 2166 742.9078 1483.8010 1483.8610 -0.0599 0 U R.IQSIAPSLQVITSK.Q**<br>**2153 – 2166 742.9078 1483.8010 1483.8610 -0.0599 0 U R.IQSIAPSLQVITSK.Q**<br>**2153 – 2166 742.9268 1483.8390 1483.8610 -0.0219 0 U R.IQSIAPSLQVITSK.Q**<br>**2284 – 2301 643.6200 1927.8382 1927.9276 -0.089 0 UK.VEVFEHAVNNTAGDDLAK.L**<br>**MS/MS Fragmentation of LFDAPEAPLPSR**<br>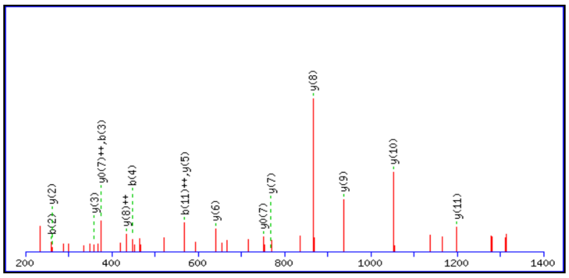

Table S1. *Cont.*

Protein Name	[a] Peptides Matches (sequences)	[b] MS/MS Analysis
Dynamin 2	5 (3)	**1** MGNRGMEELI PLVNKLQDAF SSIGQSCHLD LPQIAVVGGQ SAGKSSVLEN<br>**51** FVGRDFLPRG SGIVTRRPLI LQLIFSKTEH AEFLHCKSKK FTDFDEVRQE<br>**101** IEAETDRVTG TNK**GISPVPI NLR**VYSPHVL NLTLIDLPGI TKVPVGDQPP<br>**151** DIEYQIKDMI LQFISRESSL ILAVTPANMD LANSDALKLA KEVDPQGLRT<br>**201** IGVITKLDLM DEGTDARDVL ENKLLPLRR**G YIGVVNR**SQK DIEGKKDIRA<br>**251** ALAAERKFFL SHPAYRHMAD RMGTPHLQKT LNQQLTNHIR ESLPALRSKL<br>**301** QSQLLSLEKE VEEYKNFRPD DPTRKTKALL QMVQQFGVDF EKRIEGSGDQ<br>**351** VDTLELSGGA RINRIFHERF PFELVKMEFD EKDLRREISY AIKNIHGVRT<br>**401** GLFTPDLAFE AIVKKQVVKL KEPCLKCVDL VIQELINTVR QCTSKLSSYP<br>**451** RLREETERIV TTYIREREGR TKDQILLLID IEQSYINTNH EDFIGFANAQ<br>**501** QRSTQLNKKR AIPNQGEILV IRRGWLTINN ISLMKGGSKE YWFVLTAESL<br>**551** SWYKDEEEKE KKYMLPLDNL KIRDVEKGFM SNKHVFAIFN TEQRNVYKDL<br>**601**RQIELACDSQ EDVDSWKASF LRAGVYPEKD QAENEDGAQE NTFSMDPQLE<br>**651** RQVETIRNLV DSYVAIINKS IRDLMPKTIM HLMINNTKAF IHHELLAYLY<br>**701** SSADQSSLME ESADQAQRRD DMLRMYHALK EALNIIGDIS TSTVSTPVPP<br>**751** PVDDTWLQSA SSHSPTPQRR PVSSIHPPGR PPAVRGPTPG PPLIPVPVGA<br>**801** AASFSAPPIP SRPGPQSVFA NSDLFPAPPQ IPSRPVR**IPP GIPPGVPSR**R<br>**851** PPAAPSRPTI IRPAEPSLLD<br>**Start-End Observed Mr(expt) Mr(calc) Delta Miss Sequence**<br>**114-123 533.3640 1064.7134 1064.6342 0.0792 0 K.GISPVPINLR.V**<br>**114-123 533.3641 1064.7136 1064.6342 0.0794 0 K.GISPVPINLR.V**<br>**230-237 439.2754 876.5362 876.4818 0.0545 0 R.GYIGVVNR.S**<br>**838-849 593.8988 1185.7830 1185.6870 0.0961 0 R.IPPGIPPGVPSR.R**<br>**838-849 593.8989 1185.7832 1185.6870 0.0963 0 R.IPPGIPPGVPSR.R**<br>**MS/MS Fragmentation of GISPVPINLR**<br>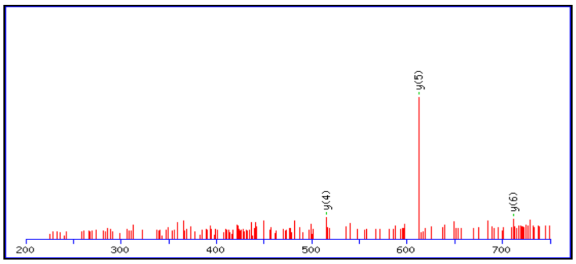
Glyceraldehyde -3-phosphate dehydrogenase	7 (4)	**1** MGK**VKVGVNG FGR**IGRLVTR AAFNSGKVDI VAINDPFIDL NYMVYMFQYD<br>**51** STHGKFHGTV KAENGKLVIN GNPITIFQER DPSK**IKWGDA GAEYVVESTG**<br>**101 VFTTMEKA**GA HLQGGAKRVI ISAPSADAPM FVMGVNHEKY DNSLKIISNA<br>151 SCTTNCLAPL AK**VIHDNFGI VEGLMTTVHA ITATQK**TVDG PSGKLWRDGR<br>**201 GALQNIIPAS TGAAK**AVGKV IPELNGKLTG MAFR**VPTANV SVVDLTCR**LE<br>**251** KPAKYDDIKK VVKQASEGPL KGILGYTEHQ VVSSDFNSDT HSSTFDAGAG<br>**301** IALNDHFVKL ISWYDNEFGY SNRVVDLMAH MASKE<br>**Start-End Observed Mr(expt) Mr(calc) Delta Miss Sequence**<br>**6-13 403.2117 804.4088 804.4243 -0.0154 0 K.VGVNGFGR.I**<br>**85-107 840.0733 2517.1981 2517.2097 - 0.01171 K.IKWGDAGAEYVVESTGVFTTMEK.A**<br>**163-186 649.5874 2594.3205 2594.3527- 0.0322 0 K.VIHDNFGIVEGLMTTVHAITATQK.T**<br>**163-186 649.5874 2594.3205 2594.3527- 0.0322 0 K.VIHDNFGIVEGLMTTVHAITATQK.T**<br>**163-186 649.5876 2594.3213 2594.3527- 0.0314 0 K.VIHDNFGIVEGLMTTVHAITATQK.T**

Table S1. *Cont.*

Protein Name	[a] Peptides Matches (sequences)	[b] MS/MS Analysis
Glyceraldehyde-3-phosphate dehydrogenase	7 (4)	**201-215 706.3884 1410.7622 1410.7831 -0.0208 0 R.GALQNIIPASTGAAK.A**<br>**201-215 706.3887 1410.7628 1410.7831 -0.0202 0 R.GALQNIIPASTGAAK.A**<br>**MS/MS Fragmentation of GALQNIIPASTGAAK**<br>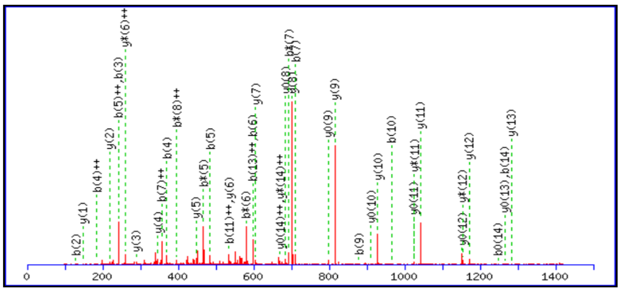
60S acidic ribosomal protein P0	3 (3)	**1** MPREDRATWK SNYFLK**IIQL LDDYPK**CFIV GADNVGSKQM QQIRMSLRGK<br>**51** AVVLMGKNTM MRKAIRGHLE NNPALEKLLP HIRGNVGFVF TKEDLTEIRD<br>**101** MLLANKVPAA ARAGAIAPCE VTVPAQNTGL GPEK**TSFFQA LGITTK**ISR**G**<br>**151** **TIEILSDVQL IK**TGDKVGAS EATLLNMLNI SPFSFGLVIQ QVFDNGSIYN<br>**201** PEVLDITEET LHSRFLEGVR NVASVCLQIG YPTVASVPHS IINGYKRVLA<br>**251** LSVETDYTFP LAEKVKAFLA DPSAFVAAAP VAAATTAAPA AAAAPAKVEA<br>**301** KEESEESDED MGFGLFD<br>**Start-End Observed Mr(expt) Mr(calc) Delta Miss Sequence**<br>**17-26 609.3789 1216.7432 1216.6703 0.0729 0 K.IIQLLDDYPK.C**<br>**135-146 657.3974 1312.7802 1312.7027 0.0775 0 K.TSFFQALGITTK.I**<br>**150-162 714.9611 1427.9076 1427.82350.0841 0 R.GTIEILSDVQLIK.T**<br>**MS/MS Fragmentation of GTIEILSDVQLIK**<br>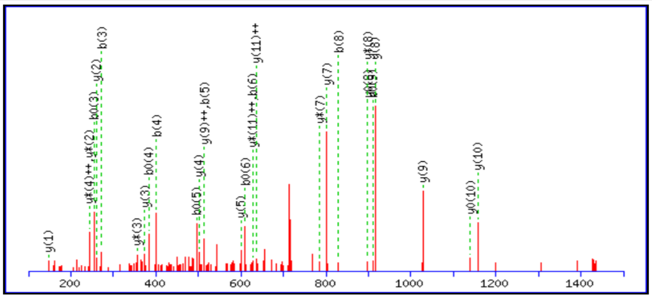
Nucleolin	8 (5)	**1** MVKLAKAGKN QGDPKKMAPP PKEVEEDSED EEMSEDEEDD SSGEEVVIPQ<br>**51** KKGKKAAATS AKKVVVSPTK KVAVATPAKK AAVTPGKKAA ATPAKKTVTP<br>**101** AKAVTTPGKK GATPGKALVA TPGKKGAAIP AKGAKNGKNA KKEDSDEEED<br>**151** DDSEEDEEDD EDEDEDEDEI EPAAMKAAAA APASEDEDDE DDEDDEDDDD<br>**201** DEEDDSEEEA METTPAKGKK AAKVVPVKAK NVAEDEDEEE DDEDEDDDDD<br>**251** EDDEDDDDED DEEEEEEEEE EPVKEAPGKR KKEMAKQKAA PEAKKQKVEG<br>**301** TEPTTAFNLF VGNLNFNKSA PELK**TGISDV FAK**NDLAVVD VRIGMTRK**FG**<br>**351** **YVDFESAEDL EK**ALELTGLK VFGNEIKLEK PKGKDSKKER DARTLLAKNL<br>**401** PYK**VTQDELK EVFEDAAEIR** LVSKDGKSKG IAYIEFKTEA DAEKTFEEKQ<br>**451** GTEIDGRSIS LYYTGEKGQN QDYRGGKNST WSGESK**TLVL SNLSYSATEE**<br>**501** **TLQEVFEK**AT FIKVPQNQNG KSKGYAFIEF ASFEDAKEAL NSCNKREIEG<br>**551** RAIRLELQGP RGSPNARSQP SKTLFVKGLS EDTTEETLKE SFDGSVRARI<br>**601** VTDRETGSSK GFGFVDFNSE EDAKAAKEAM EDGEIDGNKV TLDWAKPKGE<br>**651** GGFGGRGGGR GGFGGRGGGR GGRGGFGGRG RGGFGGRGGF RGGRGGGGDH<br>**701** KPQGKKTKFE

Table S1. *Cont.*

Protein Name	[a] Peptides Matches (sequences)	[b] MS/MS Analysis
Nucleolin	8 (5)	Start-End Observed Mr(expt) Mr(calc) Delta Miss Sequence<br>**325-333 469.2511 936.4876 936.4917 -0.0040 0 K.TGISDVFAK.N**<br>**349-362 824.8773 1647.7400 1647.7304 0.0096 0 K.FGYVDFESAEDLEK.A**<br>**349-362 824.8777 1647.7408 1647.7304 0.0104 0 K.FGYVDFESAEDLEK.A**<br>**404-420 664.6686 1990.9840 1990.9847 -0.0008 1 K.VTQDELKEVFEDAAEIR.L**<br>**411-420 589.7883 1177.5620 1177.5615 0.0006 0 K.EVFEDAAEIR.L**<br>**411-420 589.7883 1177.5620 1177.5615 0.0006 0 K.EVFEDAAEIR.L**<br>**411-420 589.7886 1177.5626 1177.5615 0.0012 0 K.EVFEDAAEIR.L**<br>**487-508 834.4281 2500.2625 2500.2584 0.0040 0**<br>**K.TLVLSNLSYSATEETLQEVFEK.A**<br>**MS/MS Fragmentation of EVFEDAAEIR**<br>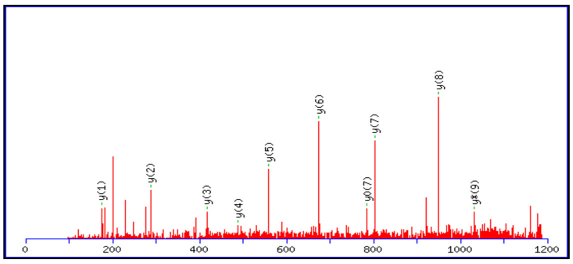
Heterogeneous nuclear ribonucleoproteins A2/B1	3 (3)	**1** MEKTLETVPL ERKKREKEQF RKLFIGGLSF ETTEESLRNY YEQWGKLTDC<br>**51** VVMRDPASKR SRGFGFVTFS SMAEVDAAMA ARPHSIDGRV VEPKRAVAR**E**<br>**101 ESGKPGAHVT VK**KLFVGGIK EDTEEHHLRD YFEEYGK**IDT IEIITDR**QSG<br>**151** KKRGFGFVTF DDHDPVDKIV LQKYHTINGH NAEVRKALSR QEMQEVQSSR<br>**201**SGR**GGNFGFGDSR**GGGGNFGPGPGSNFRGGSDGYGSGRGFGDGYNGYGGG<br>**251**PGGGNFGGSPGYGGGRGGYGGGGPGYGNQGGGYGGGYDNYGGGNYGSGNY<br>**301** NDFGNYNQQP SNYGPMKSGN FGGSRNMGGP YGGGNYGPGGSGGSGGYGGR<br>**351** SRY<br>Start-End Observed Mr(expt) Mr(calc) Delta Miss Sequence<br>**100-112 446.8949 1337.66291 337.6939 -0.0311 0 R.EESGKPGAHVTVK.K**<br>**138-147 594.8220 1187.6294 1187.6398 -0.0103 0 K.IDTIEIITDR.Q**<br>**204-213 507.2202 1012.4258 1012.4363 -0.0104 0 R.GGNFGFGDSR.G**<br>**MS/MS Fragmentation of IDTIEIITDR**<br>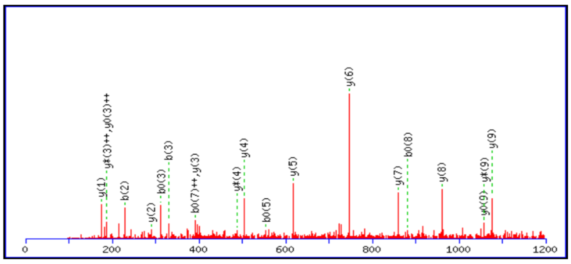
2-oxoglutarate dehydrogenase mitochondrial	9 (5)	**1** MFHLRTCAAK LRPLTASQTV KTFSQNRPAA ARTFQQIRCY SAPVAAEPFL<br>**51** SGTSSNYVEE MYCAWLENPK SVHKSWDIFF RNTNAGAPPG TAYQSPLPLS<br>**101** RGSLAAVAHA QSLVEAQPNV DKLVEDHLAV QSLIRAYQIR GHHVAQLDPL<br>**151** GILDADLDSS VPADIISSTD KLGFYGLDES DLDKVFHLPT TTFIGGQESA<br>**201** LPLREIIRRL EMAYCQHIGV EFMFINDLEQ CQWIRQKFET PGIMQFTNEE<br>**251** KRTLLARLVR STRFEEFLQR KWSSEKRFGL EGCEVLIPAL KTIIDKSSEN<br>**301** GVDYVIMGMP HRGRLNVLAN VIRKELEQIF CQFDSKLEAA DEGSGDVKYH<br>**351** LGMYHRRINR VTDRNITLSL VANPSHLEAA DPVVMGKTKA EQFYCGDTEG

Table S1. *Cont.*

Protein Name	[a] Peptides Matches (sequences)	[b] MS/MS Analysis
2-oxoglutarate dehydrogenase mitochondrial	9 (5)	**401** KKVMSILLHG DAAFAGQGIV YETFHLSDLP SYTTHGTVHV VVNNQIGFTT<br>**451** DPRMARSSPY PTDVARVVNA PIFHVNSDDP EAVMYVCKVA AEWRSTFHKD<br>**501** VVVDLVCYRR NGHNEMDEPM FTQPLMYKQI RKQKPVLQKY AELLVSQGVV<br>**551** NQPEYEEEIS KYDKICEEAF ARSKDEKILH IKHWLDSPWP GFFTLDGQPR<br>**601** SMSCPSTGLT EDILTHIGNV ASSVPVENFT IHGGLSRILK TRGEMVKNRT<br>**651** VDWALAEYMA FGSLLKEGIH IRLSGQDVER GTFSHRHHVL HDQNVDKRTC<br>**701** IPMNHLWPNQ APYTVCNSSL SEYGVLGFEL GFAMASPNAL VLWEAQFGDF<br>**751** HNTAQCIIDQ FICPGQAKWV RQNGIVLLLP HGMEGMGPEH SSARPERFLQ<br>**801** MCNDDPDVLP DLKEANFDIN QLYDCNWVVV NCSTPGNFFH VLRR**QILLPF**<br>**851** R**KPLIIFTPK** SLLRHPEARS SFDEMLPGTH FQR**VIPEDGP AAQNPENVK**R<br>**901** LLFCTGK**VYY DLTR**ERKAR**D MVGQVAITR**I EQLSPFPFDL LLKEVQKYPN<br>**951** AELAWCQEEH KNQGYYDYVK PRLRTTISRA KPVWYAGRDP AAAPATGNKK<br>**1001** THLTELQRLL DTAFDLDVFK NFS<br>**Start-End Observed Mr(expt) Mr(calc) Delta Miss Sequence**<br>**845-851 443.7497 885.4848 885.5436 -0.0588 0 R.QILLPFR.K**<br>**845-851 443.7497 885.4848 885.5436 -0.0588 0 R.QILLPFR.K**<br>**852-860 528.8093 1055.6040 1055.6743 -0.0702 0 R.KPLIIFTPK.S**<br>**852-860 528.8093 1055.6040 1055.6743 -0.0702 0 R.KPLIIFTPK.S**<br>**884-899 839.3747 1676.7348 1676.8369 -0.1021 0 R.VIPEDGPAAQNPENVK.R**<br>**884-899 839.3747 1676.7348 1676.8369 -0.1021 0 R.VIPEDGPAAQNPENVK.R**<br>**908-914 465.2073 928.4000 928.4654 -0.0654 0 K.VYYDLTR.E**<br>**908-914 465.2073 928.4000 928.4654 -0.0654 0 K.VYYDLTR.E**<br>**920-929 545.2526 1088.4906 1088.5648 -0.0742 0 R.DMVGQVAITR.I**<br>**MS/MS Fragmentation of KPLIIFTPK**<br>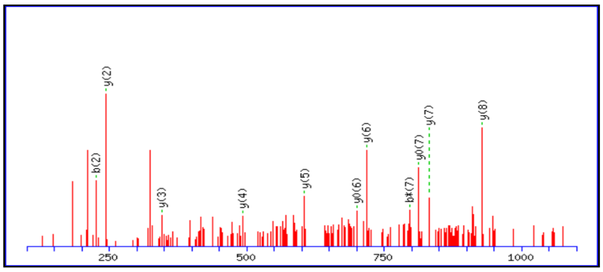
Glycosyl transferase 25 domain1	6 (6)	**1** MAAAPRAGRR RGQPLLALLL LLLAPLPPGA PPGADAYFPE ERWSPESPLQ<br>**51** APR**VLIALLA RNAAHALPTT LGALER**LRHP RERTALWVAT DHNMDNTSTV<br>**101** LREWLVAVKS LYHSVEWRPA EEPRSYPDEE GPKHWSDSRY EHVMKLRQAA<br>**151** LKSARDMWAD YILFVDADNL ILNPDTLSLL IAENKTVVAP MLDSRAAYSN<br>**201** FWCGMTSQGY YKR**TPAYIPI R**KRDRRGCFA VPMVHSTFLI DLRKAASRNL<br>**251** AFYPPHPDYT WSFDDIIVFA FSCKQAEVQM YVCNKEEYGF LPVPLRAHST<br>**301** LQDEAESFMH VQLEVMVK**HP PAEPSR**FISA PTKTPDKMGF DEVFMINLRR<br>**351** RQDRRERMLR ALQAQEIECR LVEAVDGKAM NTSQVEALGI QMLPGYRDPY<br>**401** HGRPLTKGEL GCFLSHYNIW KEVVDRGLQK SLVFEDDLRF EIFFKRRLMN<br>**451** LMRDVER**EGL DWDLIYVGR**K RMQVEHPEKA VPRVRNLVEA DYSYWTLAYV<br>**501** ISLQGAR**KLL AAEPLSK**MLP VDEFLPVMFD KHPVSEYKAH FSLRNLHAFS<br>**551** VEPLLIYPTH YTGDDGYVSD TETSVVWNNE HVKTDWDRAK SQKMREQQAL<br>**601** SREAKNSDVL QSPLDSAARD EL<br>**Start-End Observed Mr(expt) Mr(calc) Delta Miss Sequence**<br>**54-61 434.7851 867.5556 867.5905 -0.0349 0 R.VLIALLAR.N**<br>**62-76 512.2635 1533.7687 1533.8263 -0.0576 0 R.NAAHALPTTLGALER.L**

Table S1. *Cont.*

Protein Name	[a] Peptides Matches (sequences)	[b] MS/MS Analysis
Glycosyl transferase 25 domain1	6 (6)	**214-221 465.7565 929.4984 929.5334 -0.0350 0 R.TPAYIPIR.K**<br>**319-326 445.7151 889.4156 889.4406 -0.0249 0 K.HPPAEPSR.F**<br>**458-469 718.3516 1434.6886 1434.7143 -0.0257 0 R.EGLDWDLIYVGR.K**<br>**508-517 535.3163 1068.6180 1068.6543 -0.0362 1 R.KLLAAEPLSK.M**<br>**MS/MS Fragmentation of TPAYIPIR**<br>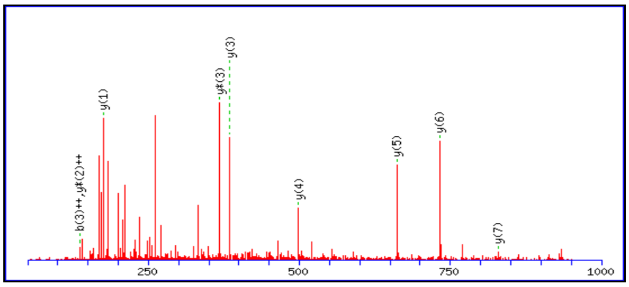
Enhancer of mRNA decapping protein 4	3 (3)	**1** MASCASIDIE DATQHLRDIL KLDRPAGGPS AESPRPSSAY NGDLNGLLVP<br>**51** DPLCSGDSTS ANKTGLRTMP PINLQEKQVI CLSGDDSSTC IGILAKEVEI<br>**101** VASSDSSISS KARGSNKVKI QPVAKYDWEQ KYYYGNLIAV SNSFLAYAIR<br>**151** AANNGSAMVR **VISVSTSER**T LLKGFTGSVA DLAFAHLNSP QLACLDEAGN<br>**201** LFVWRLALVN GKIQEEILVH IRQPEGTPLN HFRRIIWCPF IPEESEDCCE<br>**251** ESSPTVALLH EDRAEVWDLD MLRSSHSTWP VDVSQIKQGF IVVKGHSTCL<br>**301** SEGALSPDGT VLATASHDGY VKFWQIYIEG QDEPRCLHEW KPHDGRPLSC<br>**351** LLFCDNHKKQ DPDVPFWRFL ITGADQNREL KMWCTVSWTC LQTIRFSPDI<br>**401** FSSVSVPPSL KVCLDLSAEY LILSDVQRKV LYVMELLQNQ EEGHACFSSI<br>**451** SEFLLTHPVL SFGIQVVSRC RLRHTEVLPA EEENDSLGAD GTHGAGAMES<br>**501** AAGVLIKLFC VHTKALQDVQ IRFQPQLNPD VVAPLPTHTA HEDFTFGESR<br>**551** PELGSEGLGS AAHGSQPDLR RIVELPAPAD FLSLSSETKP KLMTPDAFMT<br>**601** PSASLQQITA SPSSSSSGSS SSSSSSSSL TAVSAMSSTS AVDPSLTRPP<br>**651** EELTLSPKLQ LDGSLTMSSS GSLQASPRGL LPGLLPAPAD KLTPKGPGQV<br>**701** PTATSALSLE LQEVEPLGLP QASPSRTRSP DVISSASTAL SQDIPEIASE<br>**751** ALSRGFGSSA PEGLEPDSMA SAASALHLLS PRPRPGPELG PQLGLDGGPG<br>**801** DGDRHNTPSL LEAALTQEAS TPDSQVWPTA PDITRETCST LAESPRNGLQ<br>**851** EKHKSLAFHR PPYHLLQQRD SQDASAEQSD HDDEVASLAS ASGGFGTKVP<br>**901** APRLPAKDWK TKGSPRTSPK LKRKSKKDDG DAAMGSRLTE HQVAEPPEDW<br>**951** PALIWQQQRE LAELR**HSQEE LLQR**LCTQLE GLQSTVTGHV ERALETRHEQ<br>**1001** EQRRLERALA EGQQRGGQLQ EQLTQQLSQA LSSAVAGRLE RSIRDEIKKT<br>**1051** VPPCVSRSLE PMAGQLSNSV ATKLTAVEGS MKENISKLLK SKNLTDAIAR<br>**1101** AAADTLQGPM QAAYREAFQS VVLPAFEKSC QAMFQQINDS FRLGTQEYLQ<br>**1151** QLESHMKSRK AREQEAR**EPV LAQLR**GLVST LQSATEQMAA TVAGSVRAEV<br>**1201** QHQLHVAVGS LQESILAQVQ RIVKGEVSVA LKEQQAAVTS SIMQAMRSAA<br>**1251** GTPVPSAHLD CQAQQAHILQ LLQQGHLNQA FQQALTAADL NLVLYVCETV<br>**1301** DPAQVFGQPP CPLSQPVLLS LIQQLASDLG TRTDLKLSYL EEAVMHLDHS<br>**1351** DPITRDHMGS VMAQVRQKLF QFLQAEPHNS LGKAARRLSL MLHGLVTPSL<br>**1401** P<br>**Start-End Observed Mr(expt) Mr(calc) Delta Miss Sequence**<br>**161-169 489.2541 976.4936 976.5189 -0.0253 0 R.VISVSTSER.T**<br>**966-974 570.2798 1138.5450 1138.5731 -0.0280 0 R.HSQEELLQR.L**<br>**1168-1175 463.2606 924.5066 924.5392 -0.0326 0 R.EPVLAQLR.G**<br>**MS/MS Fragmentation of VISVSTSER**

Table S1. *Cont.*

Protein Name	[a] Peptides Matches (sequences)	[b] MS/MS Analysis
Enhancer of mRNA decapping protein 4	3 (3)	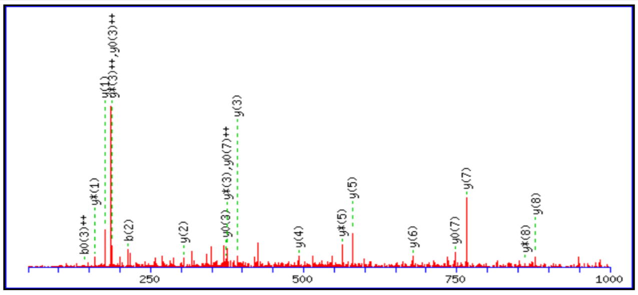
Prohibitin 2	3 (3)	**1** MAQNLKDLAG RLPAGPRGMG TALKLLLGAG AVAYGVRESV FTVEGGHRAI<br>**51** FFNRIGGVQQ DTILAEGLHF RIPWFQYPII YDIRARPRKI SSPTGSKDLQ<br>**101** MVNISLRVLS RPNAQELPSM YQR**LGLDYEE RVLPSIVNEV LK**SVVAK**FNA**<br>**151 SQLITQR**AQV SLLIRRELTE RAKDFSLILD DVAITELSFS REYTAAVEAK<br>**201** QVAQQEAQRA QFLVEKAKQE QRQKIVQAEG EAEAAKMLGE ALSKNPGYIK<br>**251** LRKIRAAQNI SKTIATSQNR IYLTADNLVL NLQDESFTRG SDSLIKGKK<br>**Start-End Observed Mr(expt) Mr(calc) Delta Miss Sequence**<br>**124-131 497.7442 993.4738 993.4767 -0.0029 0 R.LGLDYEER.V**<br>**132-142 605.8679 1209.7212 1209.7333 -0.0120 0 R.VLPSIVNEVLK.S**<br>**148-157 589.3073 1176.6000 1176.6251 -0.0251 0 K.FNASQLITQR.A**<br>**MS/MS Fragmentation of FNASQLITQR**<br>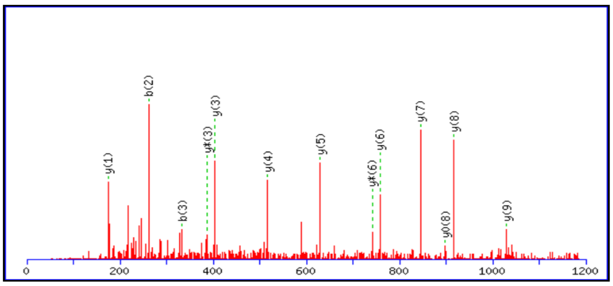

[a] Peptide matches (sequences): the number of total peptides matched with protein in the MS/MS query (the number of unique peptide sequences); [b] MS/MS analysis: the protein sequence with MS/MS identified the peptides (bold) and one exemplary MS/MS spectra of the peptide with the highest ion score.

© 2014 by the authors; licensee MDPI, Basel, Switzerland. This article is an open access articledistributed under the terms and conditions of the Creative Commons Attribution license (http://creativecommons.org/licenses/by/3.0/).
